# Is attributing smoking to genetic causes associated with a reduced probability of quit attempt success? A cohort study

**DOI:** 10.1111/j.1360-0443.2007.01937.x

**Published:** 2007-10

**Authors:** Alison J Wright, Paul Aveyard, Boliang Guo, Michael Murphy, Karen Brown, Theresa M Marteau

**Affiliations:** 1 Health Psychology, King's College London London, UK,; 2 Department of Primary Care and General Practice, University of Birmingham Birmingham, UK,; 3 Childhood Cancer Research Group, University of Oxford UK; 4 Cancer Research UK General Practice Research Group, Department of Clinical Pharmacology, University of Oxford UK

**Keywords:** Behavioural influences, causal attributions, genetic testing, perceived control, pharmacogenetic intervention, smoking cessation

## Abstract

**Aims:**

Pharmacogenetic smoking cessation interventions would involve smokers being given information about the influence of genes on their behaviour. However, attributing smoking to genetic causes may reduce perceived control over smoking, reducing quit attempt success. This study examines whether attributing smoking to genetic influences is associated with reduced quitting and whether this effect is mediated by perceived control over smoking.

**Design:**

Cohort study.

**Participants:**

A total of 792 smokers, participating in a trial of nicotine replacement therapy (NRT)-assisted smoking cessation. Participants were informed that the trial investigated relationships between genetic markers and smoking behaviour, but personalized genetic feedback was not provided.

**Setting:**

Primary care in Oxfordshire and Buckinghamshire, UK.

**Measurements:**

Perceived control over smoking and perceived importance of genetic factors in causing smoking assessed pre-quit; abstinence 4, 12, 26 and 52 weeks after the start of treatment.

**Findings:**

A total of 515 smokers (65.0%) viewed genetic factors as playing some role in causing their smoking. They had lower perceived control over smoking than smokers who viewed genetic factors as having no role in causing their smoking. Attributing smoking to genetic causes was not associated significantly with a lower probability of quit attempt success.

**Conclusions:**

Attributing smoking to genetic factors was associated with lower levels of perceived control over smoking but not lower quit rates. This suggests that learning of one's genetic predisposition to smoking during a pharmacogenetically tailored smoking cessation intervention may not deter quitting. Further research should examine whether the lack of impact of genetic attributions on quit attempt success is also found in smokers provided with personalized genetic feedback.

## INTRODUCTION

Preliminary evidence suggests that effectiveness of medication for smoking cessation may be moderated by genotype [1–3]. For example, smokers with the Asp40 variant of the mu-opioid receptor gene had double the quit rate with higher-dose nicotine replacement therapy (NRT) than with lower-dose NRT, while smokers homozygous for the more common Asn40 variant were equally likely to stop smoking regardless of NRT level [1]. Therefore, genetic testing might allow tailoring of smoking cessation therapies to maximize smokers' likelihood of quitting successfully [4]. Realizing this potential will necessitate providing smokers with information about how genetic factors contribute to their smoking. The psychological impact of providing such information requires consideration.

Leventhal's Self Regulation Model (SRM) suggests that individuals' perceptions of a health problem, including its causes, influence their perceived control over theproblem and their attempts to cope with it [5,6]. Health conditions with genetic causes are viewed as less controllable than those with behavioural or environmental causes [7]. Therefore, informing smokers that their smoking is caused partly by genetic factors may lead them to believe they have less control over their behaviour than were they not given this information. High perceived control is associated with successful recovery from addiction [8,9], so providing smokers with information about their genotype to explain the choice of cessation therapy could reduce perceived control and thereby affect adversely quit attempt success.

The SRM also suggests that changing causal beliefs about smoking by providing genetic information will influence perceptions about effective action to control the health condition. When health problems are attributed to genes, pharmacological treatments are seen as more effective [10,11] than when they are attributed to non-genetic factors. This could lead smokers who attribute their smoking to genetic causes to view nicotine replacement therapy (NRT) as an appropriate treatment, a view that may in turn increase their perceived control over stopping smoking, so countering the effect of making a genetic causal attribution.

To date, one study has examined smokers' reactions to learning of a genetic predisposition to nicotine dependence, using analogue methods [12]. Smokers read vignettes in which they were asked to imagine that they did (gene positive condition) or did not (gene negative condition) have a gene that made them more likely to be addicted to nicotine. Smokers in the gene-positive group were significantly less likely than those in the gene-negative group to say they would use willpower but more likely to say they would use bupropion to quit, as predicted by SRM. This unwillingness to use willpower might imply a reduced sense of control over smoking. However, the gene-positive and gene-negative groups had similar scores on a scale assessing perceived control, which does not accord with SRM predictions. That study was a simulation and did not focus on smokers ready to quit. In this report, we examine the effects of attributing one's smoking to genetic causal factors in real quitters.

### Hypotheses

**Figure 1:**
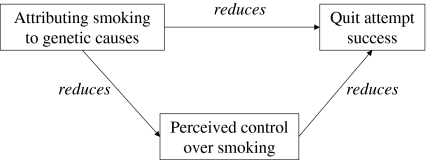
Proposed model of how the impact of viewing smoking as having a genetic cause may reduce quit attempt success

This study tests three hypotheses in a cohort of smokers making an NRT-assisted quit attempt:

1. Smokers who view genetic factors as playing a role in causing their smoking have lower perceived control over their smoking than those who do not.
2. Smokers who view genetic factors as playing a role in causing their smoking are less likely to quit successfully.
3. The effect of viewing genetic factors as causing one's smoking on quitting will be mediated by perceived control over smoking (as shown in Fig. 1).

## METHODS

### Study design

The current study involved individuals participating in the Patch in Practice (PIP) randomized controlled trial of the effects of providing moderate or low levels of support to smokers making an NRT-assisted cessation attempt (ISRCTN05689186) [13].

### Sample

Participants were eligible if they were aged 18 years and over and smoked 10 cigarettes per day or more. Participants were recruited from 26 general practices in Buckinghamshire and Oxfordshire. General practitioners (GPs) recruited patients attending for other reasons (*n* = 60, 6.5%), or patients volunteered having seen posters or heard about the study (*n* = 15, 1.6%). In some practices we wrote to every registered smoker, offering trial entry (*n* = 850, 91.9%).

Importantly, from the perspective of understanding the impact of beliefs in genetic causes of smoking on perceived control and quitting, the invitation letter described the aims of the study as being ‘to see if constitutional genetic factors affect the ability of smokers like yourself to give up using nicotine patches; to see how much nicotine replacement helps smokers to give up; and how much support from the surgery helps’. The information sheet for the trial also explained that the study would explore the role of genetic factors in smoking behaviour, stating: ‘We want to test a theory that common variations in smokers’ genes make a difference to how successful they are at using NRT'.

Potential participants were excluded only if they had contraindications to nicotine replacement therapy. Once consent was given, all participants set a quit day. Nurses encouraged participants to quit within 3–4 days of the first visit (NV1) and arranged a further visit 1 week later (NV2). At NV1, all participants were given a 15-mg nicotine patch to be worn for 16 hours per day for 8 weeks, dispensed in two packs 4 weeks apart, and completed a questionnaire.

Participants in the moderate support group had three additional contacts: a telephone call about 1 week after quit day, a nurse visit 10 days after the quit day and a third telephone call 3 weeks after NV1. All participants were scheduled for a nurse visit 4 weeks after NV1. Participants were asked to complete a questionnaire containing the psychological measures reported in this paper at NV1, and 881 of the 925 (95.2%) did so.

### Measures

#### Psychological variables

Perceived control over smoking was assessed using Velicer *et al*.'s nine-item self-efficacy for coping with tempting situations scale [14] (α = 0.84). This scale assesses smokers' perceptions of their ability to control their smoking in nine situations (e.g. ‘when I first get up in the morning’, ‘with friends at a party’) and so, despite its title, this scale can be considered to measure perceived control.

Attributing one's smoking to genetic causes was assessed using four items, developed for this study, as no suitable measures existed. Because pilot work found that smokers attributed smoking initiation to different factors than those they felt were important causes of their current smoking, the items were introduced by the statement: ‘People have different ideas about the reasons why they started smoking and the reasons why they smoke now. How important do you think the following are as causes of your smoking now?’. The four items were ‘A genetic vulnerability to nicotine addiction runs in my family’, ‘Being addicted to smoking is in my genes’, ‘I inherited a tendency to be addicted to smoking’ and ‘Addiction to nicotine runs in my family’. All items were rated on a seven-point scale with end-points of [1]‘not an important cause’ to [7]‘a very important cause’, α = 0.90. The items were interspersed with filler items about attributing smoking to other causes, such as stress and habit, results for which are not reported. Of the participants, 792 (85.6%) completed all four items. All subsequent data are derived from this subgroup of the trial participants.

#### Smoking behaviour variables

Participants' level of nicotine dependence was assessed using the Fagerström Test for Nicotine Dependence (FTND) [15]. On average, participants were moderately dependent on nicotine, with a mean (SE) FTND score of 5.1 (2.2). They also recorded the length of their longest previous quit attempt. The median (interquartile range) for this variable was 24 (177) days.

At each nurse visit, participants' exhaled carbon monoxide (CO) was measured and participants were asked whether they had smoked since the last visit and smoked during the past 7 days. Participants were telephoned at 12 weeks, 6 months and 1 year from the quit day to assess smoking status, with participants reporting abstinence sending salivary cotinine samples by post for biochemical validation. The smoking status variable used in this study was confirmed sustained abstinence. This was defined as reporting no cigarettes from NV2 (providing NV2 was undertaken 14 days or fewer from quit day) to the index time, with exhaled CO less than 10 parts per million, or salivary cotinine concentration less than 15 ng/ml on each occasion [16].

#### Participant demographics

The mean (SD) age of the participants was 43.6 (12.3) years.

### Statistical analysis

Path analysis, using Mplus [17], was used to examine whether the effect of attributing smoking to genetic causes on quitting was mediated by perceived control. In these analyses, we also controlled for the effects of nicotine dependence, longest previous quit attempt and trial arm (support level), as these may all be related to perceived control and quit attempt success. There was no evidence of multi-collinearity.

## RESULTS

The mean (SD) score on the genetic causes scale was 2.6 (1.8). However, scores on this scale were highly skewed: 277 (35.0%) participants had the lowest possible score (i.e. they responded ‘not at all an important cause’ in response to all four genetic causal attribution items). Therefore, we dichotomized this scale and used the new variable, genes viewed as possibly important in causing smoking (yes or no), in all further analyses. Differences in smoking behaviour and demographic variables between participants who viewed genes as possibly important in causing their smoking and those who did not are shown in Table 1.

**Table 1:** Differences on demographic and smoking behaviour variables between participants who did and did not view genes as possibly important in causing their smoking.

Variable	Role of genes in causing smoking?	Mean	SD	t	d.f.	P
FTND	Possibly important	5.3	2.1	−4.43	776	<0.001
	Not at all important	4.6	2.2			
Longest previous quit attempt (days)	Possibly important	220	628	0.27	779	0.79
	Not at all important	232	498			
Age (years)	Possibly important	45	12.1	−3.43	787	0.001
	Not at all important	42	11.9			

		*% female*	*n female*	*χ*^*2*^	*d.f.*	*P*
Gender	Possibly important	51.7	266	0.044	1	0.82
	Not at all important	52.7	146			

FTND: Fagerström Test for Nicotine Dependence.

The first hypothesis was that smokers who viewed genetic factors as playing a role in causing their smoking would have lower perceived control over their smoking than those who did not. Participants who viewed genes as a possible cause of their smoking reported significantly lower perceived control over smoking [mean (SD) = 2.59 (0.67)] than those who did not [mean (SD) = 2.76 (0.68)]. This difference was significant, controlling for FTND and length of previous quit attempt, *F*_1,737_ = 6.91, *P* = 0.009.

Secondly, we predicted that smokers who viewed genetic factors as playing a role in causing their continued smoking would be less likely to quit. People who viewed genes as important were less likely to quit at all time-points, but the effects were small and were not statistically significant (Table 2).

**Table 2:** Quit rates according to whether participants perceived genes as a possible cause of their smoking.

	Genes not important, n = 277		Genes possibly important, n = 515			
Time point	% quit	n	% quit	n	Unadjusted odds ratio (95% CI) for the effect of perceiving genes as possibly important on quitting	Adjusted odds ratio (95% CI) for the effect of perceiving genes as possibly important on quitting*
1 week	39.0	108	37.5	193	0.94 (0.70–1.27)	0.95 (0.64–1.30)
4 weeks	25.6	71	22.1	114	0.83 (0.59–1.16)	0.85 (0.60–1.21)
12 weeks	16.6	46	12.8	66	0.74 (0.50–1.11)	0.72 (0.47–1.10)
26 weeks	13.4	37	9.3	48	0.67 (0.42–1.05)	0.67 (0.41–1.07)
52 weeks	10.1	28	6.6	34	0.63 (0.37–1.06)	0.61 (0.35–1.05)

* Controlling for nicotine dependence (FTND), longest previous quit attempt and trial arm.

Finally, we predicted that any effect or quitting of perceiving genes as possibly important in causing smoking on quitting would be mediated by perceived control. The coefficients for the relevant path analyses are shown in Table 3. If perceived control mediated the effect of perceiving genes as important, then adding it to the model (final two columns of Table 3) should markedly reduce the association between perceiving genes as important and cessation. However, the size of the path between perceiving genes as important and quitting is barely diminished by including perceived control in the model. This was confirmed by the formal test of mediation. Neither the total, nor the indirect, effects of viewing genes as a cause of smoking on quitting were significant at any time-point. We can therefore conclude that although perceiving genes as a possible cause of smoking decreased perceived control, it had no effect on quitting. Therefore, the third hypothesis was not confirmed.

**Table 3:** Regression coefficients for mediation analysis examining whether the impact of viewing genes as a possible cause of smoking on quitting is mediated by perceived control over smoking.*

	Paths between variables in the mediation analysis							
	
Time-point	Genes as possible cause → perceived control β (SE)	P	Perceived control†→ quit β (SE)	P	Genes as possible cause → quit, perceived control not in the model β (SE)	P	Genes as possible cause → quit, adding perceived control to the model β (SE)	P
1 week	−0.174 (0.051)	0.001	0.194 (0.118)	0.101	−0.053 (0.161)	0.742	−0.028 (0.162)	0.860
4 weeks	−0.174 (0.051)	0.001	0.182 (0.134)	0.174	−0.163 (0.181)	0.368	−0.139 (0.182)	0.445
12 weeks	−0.174 (0.051)	0.001	−0.008 (0.163)	0.638	−0.330 (0.217)	0.130	−0.331 (0.219)	0.130
26 weeks	−0.174 (0.051)	0.001	−0.115 (0.183)	0.529	−0.409 (0.241)	0.090	−0.424 (0.243)	0.080
52 weeks	−0.174 (0.051)	0.001	−0.257 (0.217)	0.236	−0.053 (0.280)	0.072	−0.536 (0.281)	0.056

* Controlling for trial arm, FTND and length of longest previous quit attempt.

† Perceived control measured on a five-point scale with a mean (SD) of 2.64 (0.70).

## DISCUSSION

As predicted, smokers who viewed genes as a possible cause of their smoking perceived lower control over their smoking. However, contrary to the second hypothesis, these participants were not significantly less likely to quit successfully. The final hypothesis of this study was that the lower quit attempt success experienced by smokers who perceived genes to be a possible cause of their smoking would be mediated by these smokers feeling that they had less control over their smoking. However, given that smokers who perceived genes to be a possible cause of their smoking did not have lower quit attempt success, it is not surprising that such mediation was not demonstrated.

Previous research suggested that attributing a condition to genetic causes would be associated with lower perceived control over the condition [7]. We replicated this in the context of smoking behaviour. This finding contrasts with Wright *et al*.'s study [12] of no difference in perceived control over smoking between smokers asked to imagine they did or did not receive a positive result of a genetic test for vulnerability to nicotine dependence. However, the effect size in this study was similar (d = 0.25, 95% CI 0.10–0.40) to that in the Wright *et al*. study (d = 0.33, 95% CI −0.01–0.67), so the main reason for this is probably that the Wright *et al*. study lacked power to observe this relatively small effect. Given the magnitude of the effect, the difference in perceived control may not be clinically significant.

Finally, the present study examined the effect of genetic causal attributions on behaviour, but did not find that people who viewed their smoking as possibly caused by genes were less likely to quit successfully. Perceived control did not predict quitting in this study, whereas results of other studies reported this association [8]. There are several possible reasons for this discrepancy. First, in the current study all participants received NRT and support from their practice nurse. These may have helped participants to overcome their lack of perceived control and quit successfully. Perceived control may predict quitting more strongly when pharmacotherapy is not made available. Secondly, some evidence suggests that very high levels of self-efficacy for quitting, a construct closely related to perceived control, may have a detrimental effect on quit attempt success. Such quitters might neglect to use of coping skills and put themselves in high-risk situations [18]. For this subgroup of smokers there would be a negative association between perceived control and quit attempt success, while for other, less confident smokers, the association would be positive. Combining those two groups may lead to the two contrasting associations cancelling each other out, resulting in no significant observed association between perceived control and quit attempt success. However, we tested for this and found no evidence in support of a negative association between perceived control and quitting in smokers with the highest levels of perceived control. Thirdly, although some studies have shown associations between the perceived control over smoking and quitting [19,20], these studies did not use biochemical validation of smoking status, unlike the present study. This may have caused biased estimates of the relationship between scores on this scale and quit rates. Individuals with higher scores might have been more likely to report that they were non-smokers because their greater perceived control led to their perceiving any lapses as temporary, thus reporting themselves to be non-smokers. Finally, there may be specific psychometric issues with the particular scale used in this study. Another study using the same instrument also found no association between perceived control and quitting [21].

### Implications for theory, research and practice

Contrary to prediction, there was no association between smokers viewing genes as a possible cause of their behaviour and a reduced likelihood of smoking cessation. One explanation for this is that attributing smoking to genetic causes does not influence quit attempt success. In a study of individuals with familial hypercholesterolaemia, those who received mutation-positive results of genetic testing were just as likely to perform risk-reducing behaviours as individuals who received mutation-negative results or who were not offered testing [11]. Therefore, contrary to speculation, making genetic attributions may not cause fatalism and deter behaviour change.

Secondly, the reasons for which smokers in the present study attributed their smoking to genetic causes are unknown. Smokers who believed genes were a possible cause of their smoking may not have had genotypes that predisposed them to difficulties quitting, and so quit successfully. Alternatively, if smokers' attributions to genetic causes do reflect accurately the genetic influence on their smoking behaviour, then controlling for nicotine dependency (which somewhat reflects the individual's genotype) may have attenuated the relationship between attributions to genetic causes and quit attempt success. However, FTND score was only associated significantly with quit attempt success at 1 and 4 weeks of follow-up, and the associated odds ratios were relatively small.

Recent formulations of the Self-Regulation Model [6] suggest that it would have been useful to measure perceptions of treatment control—the belief that medications or health professionals can successfully control a health condition—in addition to perceived personal control over smoking. The lower perceived personal control reported by smokers who attributed their smoking to genetic causes might be merely a corollary to their having increased perceptions of treatment control. Smokers who thought genes were a possible cause of their smoking may have felt that NRT was more appropriate than willpower for helping them stop smoking [12]. Therefore, smokers in the present study might have thought their use of NRT was an important factor in controlling the success of their cessation attempt and so were able to quit successfully while using nicotine replacement products.

In terms of whether pharmacogenetic smoking cessation strategies are likely to be effective, this study's results suggest that viewing genes as a cause of one's smoking is not necessarily a barrier to successful quitting. However, it is important to remember that participants in this study were not given personalized information about the role of genetic factors in their smoking. Whether the provision of personalized genetic feedback leads to fatalism, and so reduced quit-attempt success, remains unknown. Personalized genetic feedback may not have the detrimental effects on behaviour change. Instead of perceiving diminished control over the problem, individuals may instead make extra efforts to control it through risk-reducing behaviour. In support of this, a study of individuals given either a genetic or a non-genetic diagnosis of familial hypercholesterolaemia showed that genetic feedback did not reduce perceived control over the condition or over risk-reducing behaviour [11].

### Strengths, limitations and suggestions for further research

This is the first study to examine the relationship between attributing smoking to genetic causes and biochemically validated abstinence. It is also the first study to examine the relationship between viewing genes as a cause of smoking and perceived control over smoking in a group of smokers who are ready to quit. It can inform studies of the impact of providing smokers with pharmacogenetically tailored smoking cessation interventions. This study benefits from using a large sample of smokers recruited from primary care who are likely to be representative of British smokers who might volunteer for trials of pharmacogenetic treatment strategies. However, the participants are not representative of the general population of British smokers, as treatment services see more dependent smokers. Also, the recruitment materials mentioned that one of the study's aims was to examine if genetic factors affect smokers' ability to quit using nicotine patches, which may have resulted in the preferential recruitment of smokers concerned about genetic causes of their smoking behaviour. However, these are exactly the smokers we might expect to respond to invitations for pharmacogenetic cessation interventions.

The present study has some limitations. It is not possible to establish the causal direction of the links between perceiving genes as a possible cause of one's smoking and perceived control, as both were measured concurrently and neither was manipulated experimentally. It would also be informative to explore smokers' beliefs about genetic influences on smoking and smoking cessation more fully. For example, do smokers who believe that genetic factors influence their smoking also believe that they will therefore have greater than average difficulty in changing their behaviour? Are smokers who believe genes influence their smoking more likely to view pharmacological cessation therapies as effective? Randomized trials of the effects of providing pharmacogenetic feedback during smoking cessation attempts including more complete measurement of smokers' beliefs regarding the influence of genetic factors of quitting, such as the recently initiated Genetic Risk and Behaviour change (GRaB) trial (ISRCTN14352545), are needed. Future research could also examine how well smokers' beliefs about the importance of genetic factors to their smoking are related to their actual genotype. Individuals with strong prior beliefs that genetic factors affect their smoking may increase their self-efficacy for quitting if genetic testing shows that this is not the case. Alternatively, if this is shown to be the case and their prescription for cessation aids is tailored accordingly, their perceived control may increase. It is also possible that some smokers, even if they do have a ‘risky’ genotype, overestimate the importance of genetic factors in smoking and in their ability to quit. Such smokers may benefit from education about the multi-factorial influences on smoking and smoking cessation.

## CONCLUSIONS

Currently, little is known about the potential psychological impact of providing genetic risk information to smokers as part of pharmacogenetic smoking cessation strategies. This study found that perceiving genes as an influence on smoking was associated with slightly lower perceptions of control over smoking, but not with a reduced likelihood of stopping smoking. Further research should examine whether the lack of impact of genetic attributions on quit attempt success is also found in smokers provided with personalized genetic feedback as part of the pharmacogenetic tailoring of smoking cessation therapies.
